# Genotype/Phenotype Analyses for 53 Crohn’s Disease Associated Genetic Polymorphisms

**DOI:** 10.1371/journal.pone.0052223

**Published:** 2012-12-27

**Authors:** Camille Jung, Jean-Frédéric Colombel, Marc Lemann, Laurent Beaugerie, Matthieu Allez, Jacques Cosnes, Gwenola Vernier-Massouille, Jean-Marc Gornet, Jean-Pierre Gendre, Jean-Pierre Cezard, Frank M. Ruemmele, Dominique Turck, Françoise Merlin, Habib Zouali, Christian Libersa, Philippe Dieudé, Nadem Soufir, Gilles Thomas, Jean-Pierre Hugot

**Affiliations:** 1 Université Paris Diderot, UMR843, Paris, France; 2 UMR843, INSERM, Paris, France; 3 Service de Gastroentérologie Pédiatrique, Hôpital Robert Debré, APHP, Paris, France; 4 Service de Gastroentérologie, Hôpital Claude Huriez, Université de Lille, Lille, France; 5 Service de Gastroentérologie, Hôpital Saint-Louis, AP-HP, Université Paris- Diderot, Paris, France; 6 Department of Gastroenterology, Hôpital Saint-Antoine, AP-HP, and UPMC Univ Paris 06, Paris, France; 7 Université Paris Descartes and Service de Gastroentérologie Pédiatrique, Hôpital Necker Enfants-Malades, APHP, Paris, France; 8 Service de Gastroentérologie Pédiatrique, Hôpital Jeanne de Flandre, Université de Lille 2, Lille, France; 9 CEPH/Fondation Jean Dausset, Paris, France; 10 Centre D’Investigation Clinique 9301, Hôpital Cardiologique, INSERM, Lille, France; 11 Université Paris Diderot and Service de Rhumatologie, Hôpital Bichat, Paris, France; 12 Université Paris Diderot and Service de Biochimie Génétique, Hôpital Bichat, Paris, France; University of Illinois at Chicago, United States of America

## Abstract

**Background & Aims:**

Recent studies reported a role for more than 70 genes or loci in the susceptibility to Crohn’s disease (CD). However, the impact of these associations in clinical practice remains to be defined. The aim of the study was to analyse the relationship between genotypes and phenotypes for the main 53 CD-associated polymorphisms.

**Method:**

A cohort of 798 CD patients with a median follow up of 7 years was recruited by tertiary adult and paediatric gastroenterological centres. A detailed phenotypic description of the disease was recorded, including clinical presentation, response to treatments and complications. The participants were genotyped for 53 CD-associated variants previously reported in the literature and correlations with clinical sub-phenotypes were searched for. A replication cohort consisting of 722 CD patients was used to further explore the putative associations.

**Results:**

The *NOD2* rare variants were associated with an earlier age at diagnosis (p = 0.0001) and an ileal involvement (OR = 2.25[1.49–3.41] and 2.77 [1.71–4.50] for rs2066844 and rs2066847, respectively). Colonic lesions were positively associated with the risk alleles of *IL23R* rs11209026 (OR = 2.25 [1.13–4.51]) and 6q21 rs7746082 (OR = 1.60 [1.10–2.34] and negatively associated with the risk alleles of *IRGM* rs13361189 (OR = 0.29 [0.11–0.74]) and *DEFB1* rs11362 (OR = 0.50 [0.30–0.80]). The *ATG16L1* and *IRGM* variants were associated with a non-inflammatory behaviour (OR = 1.75 [1.22–2.53] and OR = 1.50 [1.04–2.16] respectively). However, these associations lost significance after multiple testing corrections. The protective effect of the *IRGM* risk allele on colonic lesions was the only association replicated in the second cohort (p = 0.03).

**Conclusions:**

It is not recommended to genotype the studied polymorphisms in routine practice.

## Introduction

Crohn’s disease (CD) is a complex genetic disorder resulting from the interplay between environmental and genetic risk factors. To date, more than 70 genes or loci have been associated with the susceptibility to CD, each with a small individual effect on disease risk [1]–[20]. A strong overlap with genes predisposing to ulcerative colitis was found among them [21]. The identified genes belong to several biological pathways including bacterial recognition/innate immunity/autophagy (*NOD2*, *ATG16L1*, *IRGM*), adaptative immunity (*IL23R*, *CCR6*, *IL12B*, *JAK2*, *STAT3*) and inflammatory response (*TNFSF15*, *PTPN2*). For some associated genes, there is no clear established role (*ZNF365*, *CCNY*), and even some CD-associated polymorphisms are located in gene desert regions.

CD is a heterogeneous disorder with different clinical presentations. The disease may occur at any point from childhood to old age, and some CD cases require surgery and/or immunosuppressive therapy while others are characterized by rare relapses that are easily treated with anti-inflammatory drugs. Complications such as severe colitis, strictures and fistulas are common but not constant. Finally, the responses to treatments vary between patients. Unfortunately, to date, only limited parameters are available to define the early clinical course of CD [22]. As a result, some patients can be undertreated while others can be exposed to the side effects of drugs without clear benefit.

Genetic factors can be regarded as good candidates for classifying patients in terms of disease location, severity, complications, extra-intestinal manifestations and drug response/toxicity. The impact of *NOD2* mutations has been extensively studied and its associations with a young age of onset, an ileal location and complicated behaviours are well established. However, *NOD2* status is not sufficient by itself to influence clinical practice [23], [24]. Less consistent associations have been reported for other CD susceptibility genes, but again no recommendations have been formulated (for a review see [24]). In contrast, a few studies have investigated a large number of CD susceptibility alleles with special attention being paid to clinical items able to impact upon clinical practice. Henckaerts et al. identified positive associations between rs1363670 (close to *IL12B*), rs12704036 (in a gene desert region) and rs 6908425 (*CDKAL1*) polymorphisms and disease behaviour [25]. However, these results remain to be confirmed. The aim of this study was to further investigate 53 CD-associated variants in a large cohort of CD patients with detailed medical records in order to determine the genotype/phenotype relationships.

## Subjects and Methods

### Ethic Statements

The study received approval from the French national ethic committee (Hôpital Saint Louis, Paris, France) and all participants signed an informed consent form.

### Patients

Six paediatric and adult gastroenterology tertiary centres recruited 798 CD cases as defined by the Lennard-Jones criteria [26]. The patients were included if the diagnosis of CD was made at least one year before inclusion and if they had attended continuous follow-up visits in the reference centres. More than 94% of the patients included had European origins while the others mainly originated from North Africa. The replication cohort consisted of 722 familial CD cases recruited through a European consortium [3]. A panel of 960 healthy blood donors without personal or familial history of inflammatory disorders [27] was also genotyped to evaluate the association of the studied SNPs with CD in the French population.

### Phenotypic Data Recorded

Clinical, endoscopic, radiological and histological data were retrospectively collected on a standardized questionnaire by clinical research assistants, validated by the referring expert gastroenterologists of the patients and reviewed by the data managers of the study. The load factor of each item was up to 95%. In order to validate the quality of the data, the outlier values were searched for and verified and a sample of 200 questionnaires was checked twice. The items recorded included sex, date of birth, smoking habits (patients were classed as smokers in the case of any smoking habit) and presence of granulomas. Familial history of Inflammatory Bowel Diseases (IBD) was defined by a reported diagnosis of IBD in one or more first or second degree relatives.

The involvement of the digestive tract was registered at diagnosis and at the end of follow-up (cumulative locations) for the esophagus, stomach, duodenum, jejunum, proximal ileum, distal ileum, colon and rectum. Involvement was defined by macroscopic lesions. Disease behaviour’ was classified according to the Montreal classification at last follow-up (B1: non-stricturing, non penetrating disease; B2: stenosing behavior, B3 penetrating disease excluding perianal disease).

The use, response and side effects were recorded for corticosteroids, azathioprine/6-mercapopurine, methotrexate, infliximab and enteral feeding. Steroid exposure was classified as being mild (less than 2 months per year), moderate or frequent (more than 6 months per year). Patients who relapsed when the steroid dosage was tapered or within 3 months after treatment ended were defined as steroid dependent. There is no consensus definition of steroid resistance [28] and patients who showed no response after 2 weeks of full steroid doses (at least 1 mg/kg/d in children and 60 mg/d in adults) were defined as corticosteroid resistant. For the other drugs, treatment failure was determined by the IBD gastroenterologist. Indications of surgery were classed as follows: penetrating complications, stenosing disease, failure of medical treatment and anal surgery. Total gut resection was classified as major resection (total colectomy or small bowel resection >50 cm), limited resection or no resection. Proctologic surgery included all surgical procedures for perianal CD performed under general anaesthesia and with therapeutic actions.

Malnutrition (defined by a loss of two standard deviations on the weight curve in children or by the need of artificial nutrition in adults), bleeding requiring blood transfusion, severe colonic attacks and extra-intestinal manifestations were noted. Patients were classified as never hospitalized (excluding the initial diagnosis procedure and single day hospitalizations), frequently hospitalized (more than once a year) or intermediate. The evolution types were defined as frank relapses and remissions; chronic continuous evolution and other.

### Genotyping

All patients and controls were genotyped for 53 reported CD susceptibility Single Nucleotide Polymorphisms (SNPs, Table 1) using an AB17900HT Sequence detection system Illumina GoldenGate assay (Illumina, San Diego, CA) by the Centre National de Génotypage (CNG, Evry, France) or by the Integragen company (Evry, France). This set of SNPs was retained on the basis of the available literature and corresponded to the 50 SNPs with the highest published OR and the three main *NOD2* associated variants. The genotyping rate of each SNP was higher than 90% (Table 1). All genotyped SNPs were in Hardy-Weinberg equilibrium in patients and in controls. No major discrepancies were observed between our calculated allele frequencies and those published in the SNP database. For rs916977, we observed a minor allele frequency of 0.25, whereas the published estimates varied between 0.13 and 0.42. The at-risk alleles were defined as the alleles previously associated with CD in the literature. For clarity, the nucleotides defining the risk alleles are indicated in table 1 and between brackets in the text.

**Table 1:** Genetic polymorphisms studied.

SNP	chromosome	candidate genes	Major/Minor allele	risk allele	MAF (Controls)	MAF (CD)	p-value	OR	[CI 95%]	genotyping succes rate (%)
**rs11209026 ** [**4**]	1p31	IL23R	G/A	G	0.08	0.02	**1.94E−11**	**0.30**	**[0.20–0.43]**	100
**rs2476601 ** [**2**]	1p13	PTPN22	G/A	G	0.08	0.05	**1.2E−03**	**0.62**	**[0.46–0.83]**	99
rs2274910 [2]	1q23	ITLN1/CD244	C/T	C	0.34	0.31	1.9E**−**01	0.90	[0.77–1.05]	97
**rs9286879 ** [**2**]	1q24	FASLG/TNFSF18/FNFSF4	A/G	G	0.26	0.3	**2.1E−02**	**1.20**	**[1.02–1.41]**	96
rs12035082 [7]	1q24	FASLG/TNFSF18/FNFSF4	C/T	T	0.45	0.46	2.7E**−**01	1.08	[0.94–1.24]	98
rs10801047 [6]	1q31	?	T/A	A	0.1	0.08	1.6E**−**01	0.83	[0.65–1.07]	98
rs11584383 [2]	1q32	C1orf106/KIF21B	T/C	T	0.24	0.23	5.5E**−**01	0.95	[0.80–1.12]	98
rs10733113 [14]	1q44	NLRP3	G/A	G	0.14	0.15	4.3E**−**01	1.08	[0.88–1.32]	98
**rs2241880 ** [**1**]	2q37	ATG16L1	A/G	G	0.45	0.4	**3.1E−03**	**0.80**	**[0.70–0.93]**	98
rs3197999 [7]	3p21	MST1/GPX1/BSN	C/T	T	0.26	0.29	8.0E**−**02	1.15	[0.98–1.35]	94
rs16853571 [5]	4p12	PHOX2B	C/G	C	0.07	0.06	6.1E**−**01	0.93	[0.70–1.22]	98
**rs17234657 ** [**11**]	5p13	PTGER4	T/G	G	0.12	0.15	**1.4E−02**	**1.28**	**[1.05–1.56]**	98
**rs2631367 ** [**9**]	5q31	SCL22A4	G/C	C	0.49	0.55	**1.0E−03**	**1.27**	**[1.10–1.47]**	90
**rs1050152 ** [**9**]	5q31	SCL22A5	C/T	T	0.43	0.48	**3.6E−03**	**1.23**	**[1.07–1.42]**	98
**rs2188962 ** [**2**]	5q31	SLC22A4/A5. IRF1. IL3	C/T	T	0.43	0.48	**3.0E−03**	**1.23**	**[1.07–1.42]**	98
**rs13361189 ** [**6**]	5q33	IRGM	T/C	C	0.13	0.18	**6.6E−05**	**1.46**	**[1.21–1.77]**	100
**rs10045431 ** [**2**]	5q33	IL12B	C/A	C	0.28	0.25	**3.6E−02**	**0.84**	**[0.71–0.99]**	98
rs6908425 [2]	6p22	CDKAL1	C/T	C	0.21	0.19	0.15	0.89	[0.76–1.04]	97
rs7746082 [2]	6q21	PRDM1	G/C	C	0.25	0.28	9.3E**−**02	1.15	[0.97–1.35]	97
rs2301436 [2]	6q27	CCR6	G/C	C	0.47	0.48	0.32	1.07	[0.94–1.21]	98
rs1456893 [2]	7p12	IKZF1/ZPBP/FIGNL1	A/G	G	0.3	0.29	5.2E**−**01	0.95	[0.81–1.11]	98
rs2160322 [20]	7q21	MAGI2	C/G	C	0.39	0.36	8.4E**−**02	0.88	[0.76–1.01]	97
rs4728142 [17]	7q32	IRF5	G/A	A	0.43	0.43	8.5E**−**01	1.01	[0.87–1.17]	98
rs11362 [16]	8p23	DEFB1	G/A	G	0.42	0.42				98
**rs1551398 ** [**2**]	8q24	?	A/G	A	0.42	0.39	**4.7E−02**	**0.86**	**[0.74–0.99]**	98
rs10758669 [2]	9p24	JAK2	A/C	C	0.35	0.38	9.6E**−**02	1.13	[0.97–1.31]	98
**rs4263839 ** [**2**]	9q32	TNFSF15	G/A	G	0.31	0.27	**1.7E−02**	**0.82**	**[0.70–0.96]**	96
rs3936503 [7]	10p11	CCNY	G/A	A	0.35	0.28				98
**rs17582416 ** [**2**]	10p11	CREM	T/G	G	0.33	0.4	**7.6E−05**	**1.35**	**[1.16–1.57]**	98
rs224136 [5]	10q21	ZNF365	A/G	A	0.49	0.54	3.8E**−**01	0.92	[0.77**–**1.10]	98
**rs10761659 ** [**7**], [**8**]	10q21	?	C/T	C	0.18	0.17	**1.1E−02**	**1.20**	**[1.04–1.39]**	99
rs1248696 [10]	10q23	DLG5	C/T	T	0.08	0.09	2.7E**−**01	1.14	[0.89**–**1.45]	98
rs10883365 [6]	10q24	NKX2-3	A/G	G	0.49	0.46	0.08	0.88	[0.77**–**1.01]	98
**rs3814570 ** [**15**]	10q25	TCF7L2	C/T	T	0.28	0.24	**1.5E−02**	**0.81**	**[0.69–0.96]**	98
rs1793004 [13]	11p15	NELL1	G/C	G	0.26	0.26	7.7E**−**01	1.02	[0.87**–**1.19]	98
**rs7927894 ** [**2**]	11q13	C11orf30	C/T	T	0.36	0.42	**1.3E−03**	**1.27**	**[1.10–1.48]**	91
rs11175593 [2]	12q12	LRRK2/MUC19	C/T	T	0.02	0.03	1.3E**−**01	1.36	[0.91**–**2.03]	99
rs3764147 [2]	13q14	C13orf31	A/G	G	0.24	0.27	1.1E**−**01	1.14	[0.97**–**1.34]	98
**rs916977 ** [**7**]	15q13	HERC2	G/A	A	0.3	0.25	**2.6E−03**	**0.78**	**[0.66–0.91]**	98
**rs2066844 ** [**3**]	16q12	NOD2	C/T	T	0.07	0.14	**2.14E−11**	**2.17**	**[1.72–2.72]**	98
**rs2066845 ** [**3**]	16q12	NOD2	G/C	C	0.01	0.05	**2.6E−07**	**2.71**	**[1.82–4.02]**	97
**rs2066847 ** [**3**]	16q12	NOD2	0/insC	C	0.02	0.11	**1.3E−26**	**5.30**	**[3.78–7.42]**	97
rs8050910 [2]	16q24	FAM92B	T/G	G	0.41	0.4	4.2E**−**01	0.94	[0.81**–**1.09]	96
**rs2872507 ** [**2**]	17q21	ORMDL3/GSMDL/ZPBP2/IKZF3	G/A	A	0.41	0.45	**4.0E−02**	**1.16**	**[1.00–1.34]**	95
rs744166 [2]	17q21	STAT3/MLX	A/G	A	0.42	0.41	3.8E**−**01	0.93	[0.81**–**1.08]	96
rs2542151 [2]	18p11	PTPN2	T/G	G	0.15	0.15	8.8E**−**01	1.01	[0.83**–**1.22]	98
rs2305767 [19]	19p13	MYO9B	T/C	T	0.42	0.41	4.0E**−**01	0.94	[0.81**–**1.08]	98
rs2315008 [12]	20q13	TNFRSF6B	C/A	C	0.28	0.26	1.6E**−**01	0.89	[0.75**–**1.04]	98
**rs1736135 ** [**2**]	21q21	?	T/C	T	0.45	0.41	**2.1E−02**	**0.84**	**[0.73–0.97]**	97
**rs762421 ** [**2**]	21q22	ICOSLG	A/G	G	0.35	0.4	**1.7E−02**	**1.19**	**[1.03–1.38]**	96
rs2836878 [12]	21q22	PSMG1	C/T	C	0.25	0.25	0.90	0.99	[0.85**–**1.14]	98
rs35873774 [18]	22q12	XBP1	A/C	T	0.04	0.04	6.8E**−**01	1.07	[0.77**–**1.49]	98
**rs4821544 ** [**5**]	22q13	NCF4	T/C	C	0.33	0.38	**4.8E−03**	**1.23**	**[1.06–1.42]**	98

The minor and major alleles are derived from public databases. Significant associations (p<0.05) are in bold. SNP: Single Nucleotide Polymorphism. MAF: minor allele frequency. CD: Crohn’s Disease. OR: Odds Ratio; CI: Confidence interval.

### Statistical Analyses

Qualitative variables were described in percentages and quantitative variables were described by their median with interquartile ranges (Q1**–**Q3). Comparisons of qualitative variables were performed using the Chi-square or Fisher’s exact tests (when n<5 in the χ^2^ contingency table). Comparisons of medians were performed using the Kruskal-Wallis or Mann-Whitney tests. The Odds-Ratio (OR) values were calculated using the logistic regression method in the univariate and multivariate analyses. Multivariate analyses took into account all of the risk factors associated (P<0.05) with the item studied in the univariate analyses, including the *NOD2* alleles. The cumulative incidences of first drug prescriptions and first surgery were drawn on Kaplan-Meier curves and compared using the log-rank test. The tests were done for each (at-risk or protective) allele of each tested marker corresponding to a recessive/dominant model of inheritance. For *NOD2*, the three rare alleles (corresponding to independent mutations) were also analyzed jointly. Statistical analyses were performed using STATA 10 statistical software (Stata Corporation, College Station, Texas, USA).

The power of the cohort to detect an association depends on the respective frequencies of the sub-phenotypes tested and of the risk alleles in the subgroups compared. As an example, the cohort was powerful enough to detect an association with an OR of 1.5 between complicated and inflammatory behaviours for risk allele frequencies ranging from 0.3 to 0.7 in the reference group (α = 0.05; ß = 0.8). We report here a comprehensive overview of the most relevant positive tests with a nominal p-value lower than 0.05, with special attention paid to items exploring the Montreal classification, the responses to treatments and severity of the disease. For this study, we explored many phenotypic items for 53 markers and we thus performed several hundred statistical tests. These tests were not always independent but the coefficient for applying the Bonferroni correction needed to be higher than 500. Under these conditions, only strong associations could remain significant. For this reason, associations with nominal p-values lower than 0.05 were further tested in the replication cohort no matter what their corrected P-values.

## Results

### Case-control Analyses

The allele frequencies of the 53 SNPs tested were compared between cases and controls. We confirmed an association between CD and 26 independent SNPs (Table 1). As expected, the most significant associations were observed for the CD susceptibility alleles with the highest reported OR, i.e. the *NOD2* mutations and the rare *IL23R* protective allele.

### Sex, Family History, Tobacco Use and Age of Onset

The description of the exploratory cohort is shown in Table 2 and Table S1. The median duration of follow-up was 7 years (Q1**–**Q3: 4**–**12.5). The significant results are summarized in Table 3. The sex ratio was not altered by the SNPs tested. The *IL23R* protective (A) allele and the *NOD2* (C) risk allele rs2066845 were associated with a positive family history of inflammatory bowel disease (Table 3). No differences were observed between smoking groups, arguing against a gene-environment interaction. As previously reported, patients with at least one *NOD2* variant had an earlier onset of disease (Table 3). No relationships were found between age at onset (or at diagnosis) and any other SNP.

**Table 2:** Main characteristics of the cohorts of Crohn’s Disease patients.

	Exploratory cohort (n = 798)		Replication cohort (n = 722)	
	Gender and age at diagnosis			
Gender	Female: 56.7%		Female: 53.7%	
Median age at diagnosis(1^st^ and 3^rd^ quartiles )	22 years(17**–**29)		22 years(16**–**30)	
	**Location of the disease**			
	At diagnosis	At follow-up	At diagnosis	At follow up
Upper digestive tract	14.5%	22%	16.2%	17.3%
Terminal ileum (TI)	70.7%	80.9%	73.3%	78%
Colon (including rectum)	73.7%	82.5%	71.2%	77%
Small bowel (excluding TI)	5.4%	10.4%	6.9%	11.6%
Rectum	31.5%	48.2%	25.2%	32.2%
Penetrating perianal disease	33.1%		32.9%	
	**Behavior of the disease**			
	At follow-up		At follow-up	
Inflammatory behavior (B1)	43.8%		29.7%	
Stricturing behavior (B2)	31.3%		22.7%	
Penetrating behaviour (B3)	24.8%		47.6%	
	**Extra-digestive manifestations** (at diagnosis)			
	22%		28.7%	
	**Treatments**			
Surgery	51.4%		55.7%	
Corticosteroids	90%		76.2%	
Immunosupressants (AZA+ MTX)	74.1%		37.4%	
Infliximab	24.9%		6.3%	
Nutritional therapy	14.2%		28.4%	
	**Smoking habits (at the time of recruitment)**			
Never	45.3%		52.8%	
Ex-smoker	29%		15.2%	
Current smoker	25.7%		32%	
	**Familial history of IBD**			
	16.9%		100%	

**Table 3:** Most significant results of the genotype/phenotype analyses obtained with the exploratory cohort.

best candidate susceptibility gene	polymorphism	associated allele	associated sub-phenotype	Nominal P-Value	Odds Ratio
*NOD2*	rs2066845	at risk allele	family history of IBD	0.034	OR = 1.80 [1.04**–**3.12]
*NOD2*	rs2066844	at risk allele	early age of onset	0.0001	NA
*NOD2*	rs2066845	at risk allele	early age of onset	0.0026	NA
*NOD2*	rs2066844	at risk allele	ileal disease	0.0001	OR = 2.25 [1.49**–**3.41]
*NOD2*	rs2066847	at risk allele	ileal disease	0.0001	OR = 2.77 [1.71**–**4.50]
*NOD2*	two SNPs versus none	at risk allele	non inflammatory disease	0.031	OR = 1.68 [1.04**–**2.69]
*NOD2*	rs2066847	protective allele	steroid dependance	0.04	OR = 0.36 [0.15**–**0.84]
*NOD2*	rs2066847	protective allele	earlier treatment with AZA	0.039	NA
*NOD2*	rs2066845	protective allele	earlier treatment with MTX	0.03	NA
*NOD2*	two SNPs versus none	at risk allele	surgery for penetrating disease	0.007	OR = 1.94 [1.19**–**3.15]
*NOD2*	two SNPs versus none	at risk allele	surgery for stenosing disease	0.005	OR = 1.90 [1.21**–**3.00]
*NOD2*	two SNPs versus none	protective	penetrating perianal disease	0.001	OR = 0.41 [0.24**–**0.67]
*NOD2*	two SNPs versus none	at risk allele	malnutrition	0.0001	OR = 3.21 [1.69**–**6.07]
*IL23R*	rs11209026	protective allele	family history of IBD	0.002	OR = 0.32 [0.15**–**0.64]
*IL23R*	rs11209026	at risk allele	colonic disease	0.021	OR = 2.25 [1.13**–**4.51]
*IL23R*	rs11209026	at risk allele	earlier surgery	0.031	NA
*IL23R*	rs11209026	protective allele	severe colonic attacks	0.009	OR = 0.16 [0.04**–**0.63]
*DEFB1*	rs11362	protective	colonic disease	0.004	OR = 0.50 [0.30**–**0.80]
*DEFB1*	rs11362	at risk allele	non inflammatory disease	0.007	OR = 1.73 [1.16**–**2.67]
*DEFB1*	rs11362	protective allele	severe colonic attacks	0.007	OR = 0.32 [0.14**–**0.74]
*IRGM*	rs13361189	protective	colonic disease	0.01	OR = 0.29 [0.11**–**0.74]
*IRGM*	rs13361189	at risk allele	non inflammatory disease	0.028	OR = 1.50 [1.04**–**2.16]
*ATG16L1*	rs2241880	at risk allele	non inflammatory disease	0.002	OR = 1.75 [1.22**–**2.53]
*CDKAL1*	rs6908425	at risk allele	better response rate to AZA/IFX	0.001	OR = 0.45 [0.29**–**0.71]
*PTPN22*	rs2476601	at risk allele	ileal disease	0.006	OR = 2.65 [1.32**–**5.30]
*CCNY*	rs3936503	at risk allele	better response rate to AZA/IFX	0.046	OR = 3.14 [1.02**–**9.71]
*6q21*	rs7746082	at risk allele	colonic disease	0.014	OR = 1.60 [1.10**–**2.34]
*10q21*	rs224136	at risk allele	better response rate to AZA/IFX	0.006	OR = 2.12 [1.24**–**3.65]

AZA = azathioprine. IFX = infliximab. NA: not applicable.

### Disease Location

For *NOD2,* the risk alleles rs2066844 (T) and rs2066847 (C) were associated with the involvement of the distal ileum. Patients carrying at least two *NOD2* mutations and with pure colonic disease were extremely rare (n = 3). The risk allele (G) of *PTPN22* (rs2476601) was associated with ileal lesions. Colonic disease (including rectum) was associated with the risk alleles of *IL23R* (G) and the chromosome 6q21 locus (T) and with the protective alleles of *IRGM* (T) and *DEFB1* (A). Analyzes performed on the bases of the Montreal classification system for disease location confirmed the associations obtained for each anatomical site but with lower P-values. After multivariate analysis, only *IL23R* and *DEFB1* remained associated with colonic disease. None of the at-risk alleles were associated with the presence of granulomas.

### Behaviour

As previously reported, patients with two *NOD2* mutations more frequently had non-inflammatory disease behaviour at diagnosis compared to patients with wild-type *NOD2*. The risk alleles of *ATG16L1* (G), *IRGM* (C) *and DEFB1* (G) were also associated with a non-inflammatory behaviour (B2+B3). These associations remained significant after multivariate analysis, suggesting that these genes acted independently to modulate disease behaviour.

### Medications

It was found that 52% of the patients were steroid-dependant and 12.5% were cortico-resistant. No associations were found with time of first steroid therapy, response to treatment or steroid exposure. **T**he patients received an immunomodulatory treatment in 74% of cases. Patients who carried the *NOD2* protective allele rs2066847 (no insertion) (respectively rs2066845, (G)) received azathioprine treatment (respectively methotrexate) earlier (p: 0.04 respectively p: 0.03). Nevertheless, these associations disappeared when the three *NOD2* mutations were taken into account. The CD risk alleles of the *CCNY* (A), *CDKAL1* (C) and 10q21 (C) loci were weakly associated with a better response to immunosupressors and/or infliximab (Table 3). Multivariate logistic regression confirmed an association with *CDKAL1* and the 10q21 risk allele (OR = 2,70[4,54**–**1,61] and OR = 2,39[1,36**–**4,18], respectively). No association was found in multivariate analysis for the response to infliximab therapy.

### Surgery

**Figure 1:**
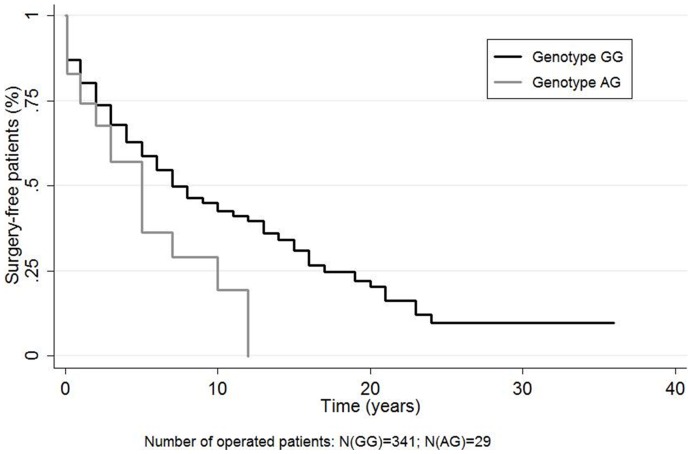
Time of the first non proctologic surgery according to IL23R rs11209026 genotype. Log Rank: P = 0.03.

The time of first non proctologic surgery did not depend on any of the SNPs tested except for the *IL23R* risk allele (G) (log-rank, p = 0.031; Fig. 1). When the analyses were performed on the subgroup of patients with pure colonic disease, the *IL23R* protective allele (A) was also predictive of an earlier surgery (log-rank, p = 0.007). Patients who carried two *NOD2* mutations had a less frequent incidence of perforating perianal disease (p = 0.001) but they were more frequently operated on for penetrating or occlusive disease. However, after adjustment for ileal location, these latter associations did not remain significant.

### Complications

Malnutrition was observed in 8.8% (n = 69) of patients at the time of diagnosis and in 10.7% (n = 84) at the end of follow-up. Patients who carried two *NOD2* mutations were more frequently exposed to this complication at diagnosis. This association was restricted to patients with ileal disease (9% for wild-type homozygotes vs. 28.3% for mutated patients, p = 0.0001) and remained significant in the subgroup of adult-CD onset. Severe colonic attacks were less frequent in patients who were homozygous for the at-risk alleles of *IL23R* (1.5% for genotype AA vs. 8.3% for genotype AG, p = 0.025) or *DEFB1* (6.3% for genotype GG vs. 0% for genotype AA, p = 0.005). No consistent associations were found with arthritis, arthralgias, mouth ulcers, cutaneous or ocular manifestations, ankylosing spondylitis, psoriasis or primary sclerosing cholangitis. No association was found with evolution type or frequency of hospitalization.

### Replication Study

As anticipated, none of the above reported associations remained statistically significant after multiple testing corrections. Therefore, we tested their relevance in the replication cohort. Because the impact of *NOD2* mutations on disease presentation was extensively studied previously (including patients from the replication cohort [28]), we only focused on the other 50 genetic markers. The exploratory and replication cohorts were not completely the same (Table 2). The main differences can be explained by the fact that the replication cohort included older patients (date of birth Q1**–**Q2**–**Q3: 1954**–**1967**–**1975 versus 1963**–**1972**–**1980, p<0.0001) with, on average, a longer follow-up (11 years). For these patients, the clinical use of immunosuppressants and biotherapies was less generalized. When comparing the patients within this cohort, we only confirmed that the risk allele (C) at rs13361189 of *IRGM* was less frequently encountered in cases of colonic disease at onset (p = 0.03).

## Discussion

Genetic studies have recently identified a large number of susceptibility genes that play a role in the predisposition to CD. The aim of this work was to assess the clinical utility of these genetic associations in routine practice. This is an important issue for the development of personalized medicine in which the genetic profile of an individual patient would help to choose optimal treatment strategies.

We analysed the clinical course of 798 CD patients from referring paediatric and adult gastroenterology centres in detail. These centres treat patients with the most severe form of the disease, as shown by the comparison between the description of the current cohort and population-based studies [29]. For example, whereas approximately only half of the patients with CD received steroid therapy at some point in the disease course and a third had steroid dependency in the population-based studies, 90% of the patients in our cohort were treated by steroids and more than 50% were steroid dependant [29]. The participant centres were used to follow the international guidelines for CD management. However, differences between therapeutic practices were likely present considering that we saw differences in the proportion of patients having received steroids, immunosupressors or surgery. However, if this heterogeneity between centres may affect disease behaviour, its impact on the here tested genotype/phenotype relationships is difficult to measure.

We studied the CD susceptibility alleles available at the time of genotyping and corresponding to the 50 alleles with the highest OR (in addition to the most common *NOD2* alleles). As shown by the case-control study, a large number of the alleles tested were positively associated with CD in our French cohort of patients, reinforcing their role in CD susceptibility. However, some alleles were not found to be associated with CD. This likely reflects the limited power of our case-control study when compared to the large meta-analyses required for identifying associations with the studied SNPs. However, some previously published CD-susceptibility alleles were not replicated, even in large cohorts of patients [19], suggesting that, in some cases, these SNPs do not indicate susceptibility to CD in all patient samples. In contrast, new SNPs have recently been added to the long list of CD susceptibility alleles [19] and additional SNPs will certainly follow. This work is thus limited by the knowledge available at the time of designing the study. However, it explored a panel of markers large enough to be representative of CD susceptibility genes. This panel contains the alleles that exhibit the strongest associations with CD. Another limitation of the study is that, except for *NOD2, IL23R, IRGM* and *ATG16L1*, CD-causing mutations have not yet been firmly established and thus the “genetic markers” tested might indirectly reflect the true causative alleles of the biological effects. However, recent in-depth sequencing of the best candidate genes does not argue for additional mutations with a larger effect in the studied candidate genes [30], [31].

The first cohort was exploratory in nature. It was used to search for putative associations that could be relevant for clinical practice, and many items of disease presentation were explored. Under these conditions, the power of the cohort to detect relevant associations should be questioned. The cohort was comparable to the cohorts followed in medium-sized adult and paediatric IBD centres. It was thus supposed to be a good tool for exploring what is relevant for “real-life”. In terms of power calculations, the cohort was large enough to detect an OR as low as 1.5 for the most common sub-phenotypes. This is in the range of what is expected to have a clinical impact. However, it is noteworthy that for less frequent sub-phenotypes (e.g. cancer or some extra-intestinal manifestations) and/or the less frequent polymorphisms, larger cohorts are required to efficiently explore this matter. In those situations, specific works focusing on specific genotype/phenotype relationships will be required, likely through large international consortia.

The exploratory cohort contained mainly Caucasian people from Europe (94%) or North Africa. Genetic heterogeneity may affect case-control studies. It is less clear that it may also affect genotype/phenotype correlation studies looking for phenotype modulating alleles. However, we performed the main analyzes again, excluding the patients with non-European ancestry. These analyzes did not significant change our conclusions (data not shown).

As previously published, the three *NOD2* SNPs were associated with ileal location and young age at onset [23], [**32**]–[**35**]. However, we did not significantly extend the spectrum of *NOD2* associated-items, confirming the conclusion that *NOD2* genotyping only has a limited impact in routine practice [25]. Among the other 50 CD susceptibility alleles studied, only a few of them were associated with some of the clinical items in the first cohort. Considering that *NOD2* is the CD susceptibility gene with the strongest effect on the phenotype, this observation suggests that genetic markers with a more limited role in CD risk may also have a limited impact on clinical presentation.

Even if some nominally significant associations were found with the first cohort, their number, nature and strength did not argue for their usefulness in clinical practice. In addition, after multiple testing corrections, none of these associations remained significant. Consequently, they would be seen by chance only. However, to better understand the significance of tests with a nominal P-value <0.05, we used a replication cohort. The replication cohort had a power comparable to the exploratory cohort but it contained familial cases only while the exploratory cohort mainly contained sporadic cases. Noteworthy, the genetic predisposition to familial and sporadic CD is the same with only limited differences observed for *NOD2* and *IL23R* (see above). In addition, there is no reason to suppose that genes involved in the modulation of CD phenotype are different in sporadic and familial CD. Thus the impact of the differences between cohorts, if any, should be limited. As a final result, we concluded that, individually, the newly identified risk alleles associated with CD do not notably contribute to the definition of clinical subgroups of patients.

In the literature, the *ATG16L1* risk allele has been inconsistently associated with ileal location, penetrating diseases and early onset [**36**]–[**42**]. We found a non-replicated association between *ATG16L1* and complicated disease behaviours. A modest association between rs4958847 of *IRGM* – which is partially correlated with rs13361189– and fistulizing behaviour/perianal fistulas has been reported [43]. The current study reports an association between the at-risk allele of rs13361189 with disease behaviour (in the first cohort) and an association between its protective allele and colonic disease at onset (in both cohorts). The last finding is in accordance with recent publications [44] but not with other ones [45], [46]. It is thus difficult to definitively retain it. Finally, even if true, this association would have only a limited impact in practice.

Most studies failed to show an association between *IL23R* and CD subphenotypes [24], [41], [**47**]–[**51**]. We found here positive associations in the first cohort but failed to reproduce them in the replication cohort. An association between the risk allele of rs11362 located in the 5′-UTR of *DEFB1* and colonic location has been published [14]. In the first cohort the *DEFB1* protective allele was inversely associated with colonic location and severe colonic relapse, whereas it was positively associated with complicated behaviours. Finally, in a previous comparable exploratory study performed on 875 CD patients, Henckaerts et al. reported associations between rs1363670 at the *IL12B* locus and a stricturing behaviour; between rs12704036 on chromosome 5q and an early penetrating behaviour and between rs6908425 in *CDKAL1* and perianal fistulas [25]. The associations obtained here were not seen in this former study while we failed to replicate Henckaerts’ results. As a whole, the comparison of our data and the literature further confirms the fact that associations between CD risk alleles and clinical sub-phenotypes are inconsistent (except for *NOD2*).

The prediction of responses to treatment is an important issue for the clinician. Unfortunately, no associations between the SNPs tested and responses to treatment and/or side effects could be obtained. The prediction of disease severity (at its best at the time of diagnosis) would also be welcome in order to propose personalized therapeutic options and to avoid rapid disease progression. As an example, a top-down strategy could be proposed to patients who are genetically at risk of developing a disabling disease while other patients with a lower risk of developing a severe course could be treated with the classic step-up strategy [52]. The definition of a severe or disabling disease is not consensual and there is a lack of validated parameters for exploring this issue [52], [53]. We thus explored a large number of clinical parameters including disease behaviour, the presence of severe colonic attacks, malnutrition, extra-intestinal manifestations, time and indications of surgery, cumulative bowel resection, and the time and use of different medications, amongst others. We also looked at the type of evolution and the frequency of hospitalization. Finally, we approached this question using a visual analogue score of severity provided by the referring clinicians of the patients (data not shown). No matter what parameter was tested, we failed to identify a relevant association between severe outcome and any of the CD susceptibility genes.

If a single allele does not predict the phenotype, it is possible that a combination of genetic variants could impact disease clinical presentation. With the exception of *NOD2* variants, no allele dosage effects were observed for any of the allele tested. The exact mechanisms by which the CD susceptibility genes contribute to this disease is not known, but many of these genes are involved in two main biological functions: i) innate immunity, including bacterial recognition and killing and ii) the Th17 pathway and inflammation. It is thus tempting to imagine an epistatic interaction between the genes involved in the same (respectively complementary) biological functions. We tested this hypothesis using the logistic regression method but we failed to identify an epistatic interaction between the genetic variants involved in innate and/or adaptive immunity in the main disease subphenotypes (data not shown). This negative result may reflect a lack of statistical power but it is in accordance with other studies that also failed to find gene-gene interactions in the susceptibility to CD [33], [36], [37], [40], [47].

It is worth noting that if CD-causing genes do not seem to play a key role in the clinical presentation of CD, the possibility that other genetic factors may contribute towards modulating the clinical presentation of the disease cannot be excluded. Indeed, disease-modifier genes might be different from disease-causing genes. A re-analysis of the large genome-wide association studies taking into account sub-phenotype classifications of the patients will help to resolve this important issue in the development of personalized medicine.

## Supporting Information

Table S1 Additional characteristics of the exploratory cohort of CD patients
(DOC)
