# Supplementary material for: Genotype/Phenotype Analyses for 53 Crohn’s Disease Associated Genetic Polymorphisms
Source: PLoS One. 2012 Dec 27;7(12):e52223. doi: 10.1371/journal.pone.0052223 (PMC3531408; doi:10.1371/journal.pone.0052223)
Supplement: Table S1 — Additional characteristics of the exploratory cohort of CD patients (DOC) [file pone.0052223.s001.doc]

**Supplementary table 1. Additional characteristics of the exploratory cohort of CD patients.**

|  | **Disease behaviour at diagnosis** | |
| --- | --- | --- |
| Inflammatory behavior (B1) | 76.6% | |
| Stricturing behavior (B2) | 14.8% | |
| Penetrating behaviour (B3) | 8.6% | |
|  | **Extra-intestinal manifestations at follow up** | |
| Cutaneous manifestations | 16% | |
| Arthritis or arthralgias | 39.4% | |
| Ocular manifestations | 6.2% | |
| Psoriasis | 6.4% | |
| Ankylosing spondylitis | 5% | |
| Primary sclerosing cholangitis | 1% | |
| Multiple sclerosis | 0.7% | |
|  | **Granulomas** | |
|  | 56% | |
|  | **Treatments** | |
| Proctological surgery | 26% | |
| Steroid-dependance | 51.6% | |
| Steroid-resistance | 32% | |
| Time under immunosuppressive therapy (in months) | 10-50.5 | |
|  | **Complications** | |
|  | At diagnosis | At follow-up |
| Severe colitis | 1.9% | 3.8% |
| Malnutrition | 8.85% | 10.7% |
| Deep anemia/severe bleeding | 2.7% | 7% |
| Cancer | 0.5% | |
|  | **Disease course** | |
| Chronic continuous | 42.3% | |
| Frank relapses and remissions | 41.2% | |
| Others | 16.5% | |
|  | **Mean (quartiles) of the visual analogical score of severity** | |
| Based on the first 3 years of the disease course | 5 (2.6-6.6) | |
| Based on all the duration of the disease course | 4.9 (2.5-6.9) | |
|  | **Frequency of hospitalizations** | |
| Never | 15.2% | |
| Intermediary | 74.7% | |
| Frequent | 10% | |
